# IL-27 Imparts Immunoregulatory Function to Human NK Cell Subsets

**DOI:** 10.1371/journal.pone.0026173

**Published:** 2011-10-19

**Authors:** Alice Laroni, Roopali Gandhi, Vanessa Beynon, Howard L. Weiner

**Affiliations:** Center for Neurologic Diseases, Brigham and Women's Hospital, Harvard Medical School, Boston, Massachusetts, United States of America; University of Nebraska Medical Center, United States of America

## Abstract

Interleukin-27 (IL-27) is a cytokine with multiple roles in regulating the immune response, but its effect on human CD56^bright^ and CD56^dim^ NK cell subsets is unknown. NK cell subsets interact with other components of the immune system, leading to cytotoxicity or immunoregulation depending on stimulating factors. We found that IL-27 treatment results in increased IL-10 and IFN-γ expression, increased viability and decreased proliferation in both CD56^bright^ and CD56^dim^ NK cell subsets. More importantly, IL-27 treatment imparts regulatory activity to CD56^bright^ NK cells, which mediates its suppressive function on T cells in a contact-dependent manner. There is growing evidence that CD56^bright^ NK cell-mediated immunoregulation plays an important role in the control of autoimmunity. Thus, understanding the role of IL-27 in NK cell function has important implications for treatment of autoimmune disorders.

## Introduction

In the last decade, evidence has accumulated about the possible role of natural killer (NK) cells, a major component of the innate immune system, in regulating the immune response by their interaction with other components of the innate and adaptive immunity [1] [2]. Human NK cells include two functionally and phenotypically distinct subsets, the CD56^bright^ and CD56^dim^ subpopulation. CD56^bright^ NK cells, which are also known as “immunoregulatory”, have limited cytotoxicity but secrete large amounts of cytokines upon stimulation [3]. In contrast, CD56^dim^ NK cells display higher cytotoxicity after stimulation and secrete less cytokines [3]. CD56^bright^ NK cells are a small percentage (5–10%) of the circulating NK population, whereas CD56^dim^ NK cells constitute the major subset (90%) [4]. Subsets of NK cells express different cytokine and chemokine receptors, which endow them with different functional and homing properties [5]. Recently, the enhancement of function of CD56^bright^ NK cells has been invoked as mechanism of action for the treatment of multiple sclerosis (MS), an autoimmune with both daclizumab and beta-interferon disease [6] [7].

IL-27 is an antigen presenting cell (APC)-derived cytokine, having a pleiotropic effect on immune cells. For example, IL-27 promotes proliferation of naïve T cells and differentiation toward a Th1 phenotype [8]. However, among Th1 polarized cells, IL-27 provides a feedback mechanism, triggering the secretion of the anti-inflammatory cytokine IL-10 [9], [10]. Moreover, IL-27 inhibits Th2 and Th17 differentiation and induces IL-10 under Th17 differentiating conditions [9]. Recently, a key role for IL-27 in the induction of IL-10 producing Tr1 cells has been reported [10], [11].

Although the immunoregulatory effect of IL-27 on T cells has been extensively studied, there are only a few reports regarding the effects of IL-27 on total NK cell phenotype and function. It has been reported that IL-27 induces the production of IFN-γ in total human NK cells and increases cytotoxicity in total mouse NK cells [12], [13] and that IL-27 receptor expression is down-modulated in total mouse NK cells after activation [14] To our knowledge, there are no studies describing the effect of IL-27 on the CD56^bright^ and CD56^dim^ NK subsets in humans.

In this study, we investigated the effect of treating CD56^bright^ and CD56^dim^ NK cell subsets with IL-27, on the phenotype, proliferation, cytokine secretion and interaction of NK cells with other cells.

## Results and Discussion

### IL-27R is differentially expressed on the surface of CD56^dim^ and CD56^bright^ NK cells

**Figure 1:**
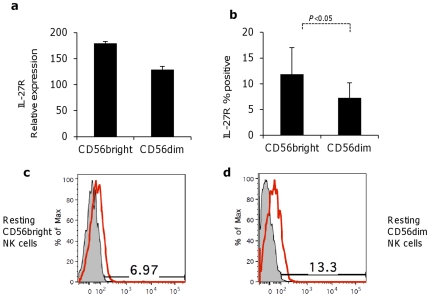
NK cells express IL-27 receptor. (a) Expression of WSX-1, subunit of IL-27R, as measured by real-time PCR in purified the CD56^bright^ and CD56^dim^ NK cells. Mean ± standard deviation of a representative experiment (of three independent experiments) is shown. (b) Expression of IL-27 receptor subunit WSX-1 on the surface of purified CD56^bright^ and CD56^dim^ NK cells, as measured by the FACS. One representative experiment of eight experiments. (c) FACS histogram showing expression of IL-27R (red profiles) on CD56^bright^ and (d) on CD56^dim^ NK cells compared to isotype control (filled grey profiles).

IL27R is comprised of two polypeptide chains, IL-27RA (WSX1) and gp130: of them, WSX1 is specific for IL-27, whereas gp130 is shared by various cytokine receptor complexes [13]. Although the expression of IL-27R on activated and non-activated NK cells has been shown previously, it was never analyzed in different subsets of NK cells, which display remarkable differences in phenotype, including receptors for cytokines [15]. Thus, the expression of IL-27R on human NK cells subsets was determined by real time PCR and by FACS analysis of surface expression of WSX1 specific subunit on both NK subsets. We found that both subsets express mRNA for IL27R (figure 1a) and express the protein on the surface with an increased expression on surface of CD56^bright^ compared to CD56^dim^ subset (p<0.05, figure 1b and figure 1c) as shown by FACS analysis.

### IL-27 increases expression of IL-10 and IFN-γ and decreases proliferation in CD56^bright^ and CD56^dim^ NK cells. Viability of NK cell subsets is increases by IL-27

**Figure 2:**
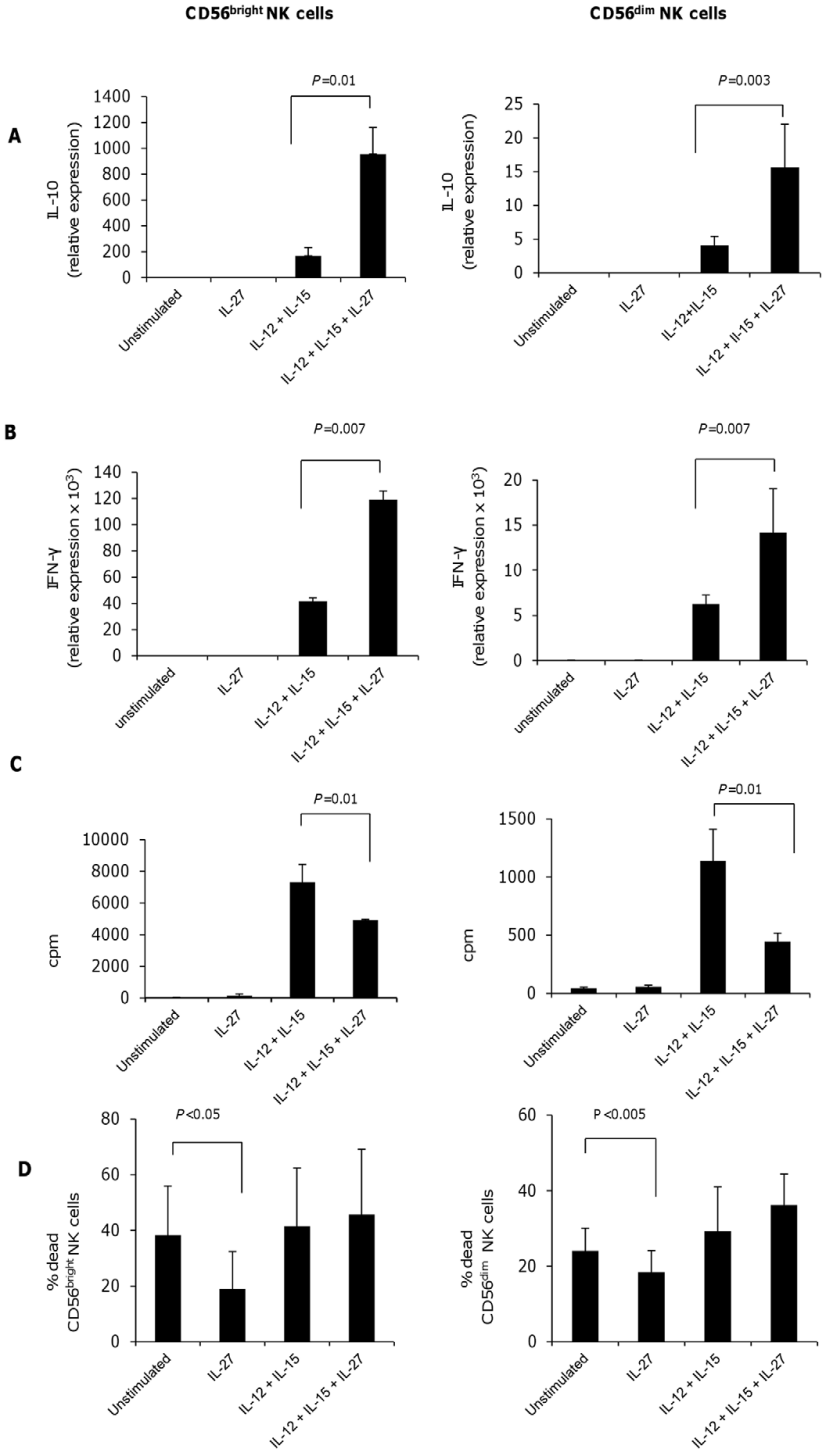
IL-27 induces the expression of IL-10 and IFN-γ in CD56bright and CD56dim NK cells. IL-27 decreases the proliferation of NK cells subsets and enhances their survival. (a) and (b) Expression of IL-10 and IFN-γ as measured by real-time PCR. Purified CD56^bright^ and CD56^dim^ NK cells were cultured for 24 hours in medium only, in presence of IL-27 only or in presence IL-12 plus IL-15 with or without IL-27. (c) Proliferation of CD56^bright^ and CD56^dim^ NK cells after culture in medium only, with IL-27 only and with IL-12 plus IL-15 with or without IL-27. Cell proliferation is shown as mean c.p.m.+s.d. in triplicate wells. (d) Viability of CD56^bright^ and CD56^dim^ NK cells as determined by the FACS after 72 hours of culture in the presence of the IL-27 compared to no stimulus, and in the presence of IL-12 plus IL-15 with or without IL-27. Dead cells were selected as AAD-positive cells. One experiment of 5–10 independent experiments is shown in each panel.

An important way by which NK cells, and especially the CD56^bright^ subset, influence innate and adaptive immune responses is the secretion of cytokines, among them IFN-γ is the most prominent, and under certain conditions, IL-10 is secreted [16] [3]. We asked whether the reported effect of IL-27 on IFN-γ secretion by NK cells occurred in both subsets, and whether IL-27 could also induce IL-10 secretion in NK cell subsets, given that this is observed following IL-27 stimulation of CD4+ T cells. We measured the expression of IFN-γ and IL-10 by real-time PCR. Although we did not observe induction of these cytokines when we stimulated NK cells with IL-27 alone (figure 2a and figure 2b) we observed increased expression of both IL-10 and IFN-γ in CD56^bright^ and CD56^dim^ NK cells stimulated with IL-12 and IL-15 and treated with IL-27 (figure 2a and 2b). It is known that IL-12 and IL-15 induce the secretion of both cytokines in NK cells [3]. The induction of a “pro-inflammatory” cytokine, such as IFN-γ, and of the immunosuppressive cytokine IL-10, may appear contradictory. However, it has been shown that in NK cells, IL-10 secretion may occur in association with IFN-γ and that IL-10^+^IFN-γ^+^ NK cells have immunosuppressive properties e.g., suppressing the secretion of the pro-inflammatory cytokine IL-12 by dendritic cells [17]. Of note regulatory Tr1 cells, induced by IL-27, also produce both IL-10 and IFN-γ [10].

We then investigated the influence of IL-27 on proliferation and viability of human NK cells [18], [19]. IL-27 alone did not induce proliferation of NK cells. We observed that IL-27 decreased IL-12 and IL-15 induced proliferation in both NK subsets (figure 2c). To exclude the possibility that this resulted from increased cell death in IL-27 stimulated NK cells, we measured viability of NK cells after the culture. We did not find decreased viability of NK cells stimulated with IL-27, IL-12 and IL-15 compared to IL-12 and IL-15 (mean viability: 55.2±20.8% and 48.3±23.5 in the two groups, figure 2d). While many cytokines have been reported to enhance proliferation of NK cells alone or in synergy with other stimuli [20], we describe for the first time that a cytokine, IL-27, has an anti-proliferative effect on NK cells. A similar anti-proliferative effect of IL-27 on activated T cells was reported previously and is thought to be critical in preventing destructive inflammation during systemic infections [21]. We also measured the influence of IL-27 alone on NK cell viability, by culturing both NK cell populations in the presence or absence of IL-27 for 72 hours. We found that NK cell viability was significantly enhanced in both NK subsets in the presence of IL-27 with no differences between CD56^bright^ vs. CD56^dim^ NK cells (figure 2d). These results demonstrate enhancement of NK cell survival in the presence of IL-27 and are consistent with reports showing that mouse NK cells, cultured in the presence of IL-27, have an increased viability [13].

### Functional effects of IL-27 on CD56^dim^ and CD56^bright^ NK cell subsets

**Figure 3:**
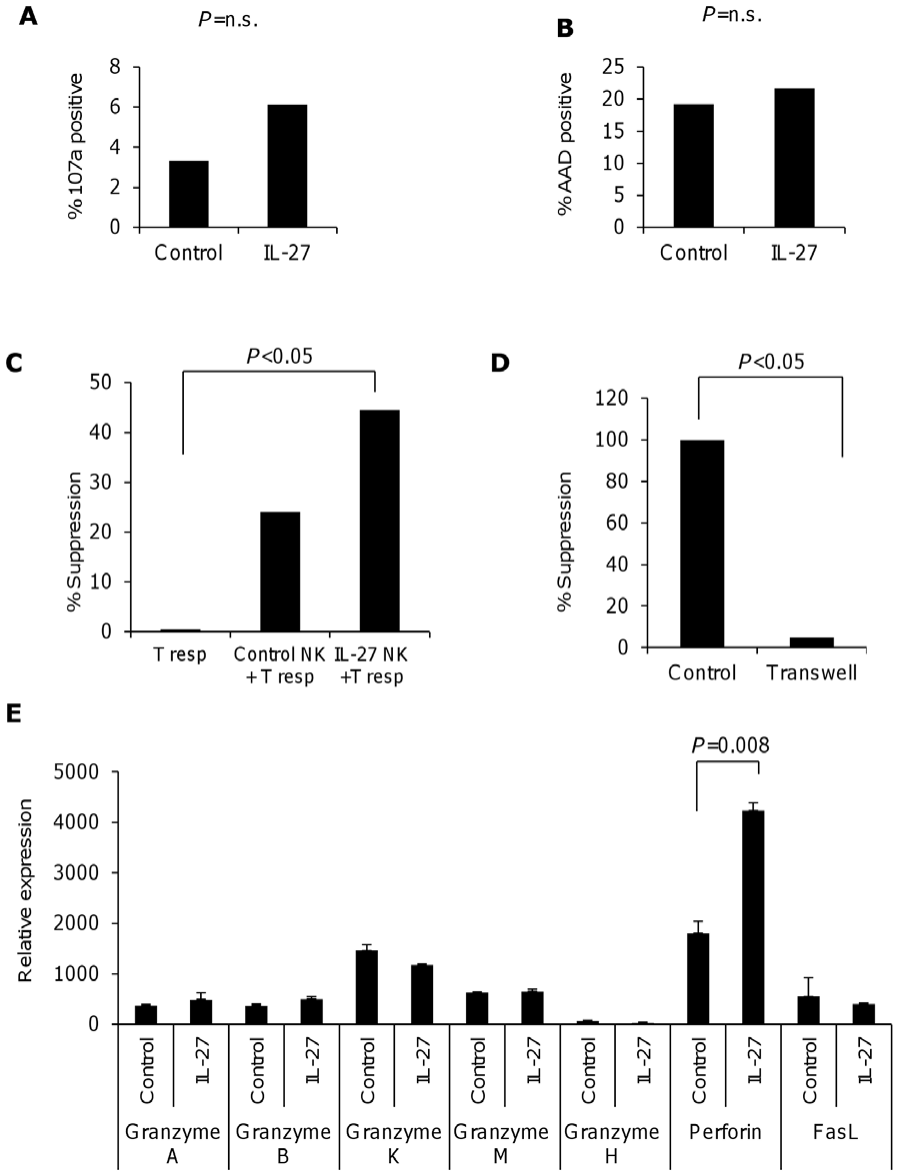
IL-27 does not induce cytotoxicity in CD56^dim^ NK cells, but imparts immunoregulatory function to CD56^bright^ NK cells. Degranulation (a) and cytotoxicity (b) of CD56^dim^ NK cells after stimulation with or without IL-27. Purified CD56^dim^ NKcells were cultured for 72 hours in the presence or absence of IL-27. Viable cells were re-sorted and cultured with or without the target cell line K562 for 4 hours or were stained for CD107a. One representative experiment is shown of 5–7 experiments. (c) Suppression assay of CD56^bright^ NK cells activated for 72 hours with IL-27. Live cells were sorted and cultured with autologous purified CD4+ T cells in the presence of anti-CD3 and anti-CD28 coated beads. Cell proliferation was assessed after five days as shown with mean+s.d. in triplicate wells, one of seven experiments is shown. (d) Suppressive activity of NK cells activated in the presence of IL-27 and incubated in contact with responder T cells (Control) or with a transwell (Transwell). (e) Expression of molecules involved in NK cell mediated cytotoxicity in CD56^bright^ NK cells treated with IL-27 after 72 hours.

Cytotoxicity is an important function of CD56^dim^ NK cells [22]. Resting CD56^dim^ NK cells are poorly cytotoxic, while activation with cytokines such as IL-2, IL-12, IL-15 and IL-21 enhances their cytotoxic features [20]. Thus, we tested IL-27-stimulated CD56^dim^ NK cells in a cytotoxic assay using K562 cells and measured both the percentage of dead cells and the percentage of degranulated (CD107a positive) CD56^dim^ NK cells as a marker for cytotoxicity. IL-27 stimulation did not enhance cytotoxicity by CD56^dim^ NK cells or influenced the degranulation as measured by CD107a expression (figure 3a and 3b).

We then evaluated the effect of IL-27 on NK cells effector functions and on their interactions with target cells. Since IL-27 is able to induce CD4+T cells with regulatory functions [10] [11], we hypothesized that IL-27 treatment might exert similar effects on NK cells. Thus, we cultured viable IL-27-treated or control CD56^bright^ or CD56^dim^ NK cells with autologous CD4+ T cells stimulated with beads coated with anti-CD3 and anti-CD28. We found that IL-27 treated CD56^bright^ NK cells demonstrated increased suppression of autologous CD4+T cells proliferation compared to untreated CD56^bright^ NK cells (figure 3c). As expected, we did not observe suppression by CD56^dim^ NK cells in the presence or absence of IL-27 (data not shown). To determine the mechanism involved in suppression mediated by IL-27-treated CD56^bright^ NK cells, co-cultures were performed in a transwell system. We found that suppression mediated by IL-27 treated NK cells was completely abolished in the transwell, suggesting that contact dependent mechanisms are required for CD56^bright^ NK cells to exert suppression (figure 3d). As NK cell cytotoxicity is primarily dependent upon cytotoxic enzymes such as perforin and granzyme B we sought to dissect which factors are involved in the suppression mediated by IL-27-treated CD56^bright^ NK cells [23]. We performed a gene expression profile of treated and untreated NK cells for molecules involved in cytotoxicity in NK cells. We observed a selective increase in the expression of perforin in IL-27-treated CD56^bright^ NK cells compared to untreated CD56^bright^ NK cells (figure 3e). This observation, together with the requirement of cell contact, might suggest a cytotoxic action of IL-27 stimulated CD56^bright^ NK cells on activated T cells; however, a direct link between perforin gene transcription induction and T cells proliferation suppression cannot be proven and should be the subject of further studies,

In summary our study shows a new immunoregulatory feature of IL-27 on important components of the innate immune system, the CD56^bright^ and CD56^dim^ NK cell subsets. There is growing evidence that NK cells, and particularly the CD56^bright^ subset, can affect the adaptive immune response through the secretion of cytokines and through a contact-dependent suppression of lymphocyte proliferation. This is of particular importance, as an increase in CD56^bright^ NK cell-mediated suppression of T cells is the presumed mechanism by which anti-CD25 monoclonal antibody (daclizumab) exerts its beneficial effects in multiple sclerosis [6] [24]. However, the mechanisms that induce these positive effects are not understood, and whether resting CD56^bright^ cells exert such effects, is not known. Here we show that treatment with IL-27 is able to induce suppressive function in CD56^bright^ NK cells and to increase the expression of IL-10, without affecting the natural cytotoxicity of CD56^dim^ NK cells towards common targets. Thus treatments which enhance IL-27 would be expected to enhance the immunoregulatory function of NK cells and could be of benefit for the treatment of autoimmune diseases, such as multiple sclerosis.

## Materials and Methods

### Subjects

Peripheral blood leukopak cells were obtained from Children's Hospital, Boston MA. The leukopak cells are obtained at the time of routine blood donation in which subjects provide written consent to have blood drawn. These procedures are in accordance with the Children's Hospital Institutional Review Board. The human cells are analyzed in our laboratory at the Brigham and Women's Hospital. Our laboratory is approved by the institutional review board at Brigham and Women's hospital for the study of human blood.

### Cell culture media, antibodies and reagents

RPMI 1640 was supplemented with 5% heat-inactivate human serum (HS), 1% nonessential amino acids, 1% sodium pyruvate, 1% HEPES buffer, 1% L- Glutamine and 1% penicillin and streptomycin (Gibco, Life technologies, Carlsbad California USA). Antibodies to CD3, CD28, IL-10, IFN-γ, CD69, CD56 and CD16 and dead cell indicator AAD were obtained from BD Biosciences. All RT-PCR primers and reagents were obtained from Applied Biosystems Life Technologies, Carlsbad California, USA. Recombinant human IL-27, IL-12 and IL-15 were obtained from R&D systems, Minneapolis, USA.

### Purification of human NK cell subsets

PBMCs were obtained by Ficoll density gradient. Total NK cells were negatively purified using a Miltenyi Biotech negative selection kit. CD3-CD56^bright^ and CD3-CD56^dim^ NK cells were sorted by FACSAria (BD Biosciences), reaching 96–98% purity in post-sort analysis.

### NK cell cultures

CD56^bright^ NK and CD56^dim^ NK cells were cultured at a concentration of 2×10^5^ cells/well in 96-well cell round-bottom culture plates in presence or absence of IL-27 (50 ng/ml) alone or in combination with IL-12 (10 ng/ml) and IL-15 (100 ng/ml). Cells were harvested 24 hours later for RNA extraction. Cell free culture supernatants were collected for cytokine analysis either by ELISA or BD cytometric bead array after 72 hours. Cells were analyzed for phenotypic markers by flow cytometry and for functional assays after 72 hours.

### Proliferation assays

Cell cultures were pulsed after 72 h of culture with ^[3H]^thymidine at 1 µCi/well for the final 18 h, harvested, and assayed for proliferation. Mean incorporation of thymidine was measured in triplicate wells and is indicated as counts per minute (cpm).

### Quantitation by real-time PCR

Total RNA was isolated from cell pellets using RNAeasy Mini Kit (Qiagen). First-strand cDNA synthesis was performed for each RNA sample from 0.5–1 µg of total RNA using Taqman reverse-transcription reagents. cDNA was amplified using sequence-specific primer and real-time PCR mix (Applied Biosystems) on ABI 7500 cycler. GAPDH gene was used as an endogenous control to normalize total RNA in each sample. All values were expressed as relative expression of gene of interest to the expression of GAPDH.

### CD107a assay and cytotoxicity assay

CD56^dim^ NK cells cultured with or without IL-27 were harvested after 72 hours, stained with 7-amino-actinomycin D (7-AAD, BD Biosciences) and sorted viable cells as 7-AAD negative cells. Viable cells were then co-cultured with or without a target cell line (K562, ATCC) at an effector-target ratio of 10∶1 for 4 hours. Half of the cells were cultured in presence of CD107a antibody (8 ul/well) or isotype control. Brefeldin A (Golgi plug, BD Bioscences) was added after an hour of culture at 0.8 ul/well. After 4 hours of culture, cells were harvested, stained with CD56 and immediately read with an LSR II flow cytometry. Half of the cells were cultured in absence of CD107a and brefeldin A and were stained, at the end of the culture, with anti-CD56 and 7-AAD, and then immediately read by flow cytometry (LSR II, BD Biosciences, CA, USA). Percentage of CD107a+ CD56+ and CD107a-CD56+ cells as well as percentage of CD56-CFSE+7AAD+ (dead target cells) and CD56-CSFE+7AAD− cells (alive target cells) were evaluated.

### Suppression assay

After 72 hrs of culture, CD56^bright^ NK cells cultured with or without IL-27 were harvested and sorted for live (7-AAD negative) cells. CD4+ T cells negatively isolated (Miltenyi kit) from the same individual were cryopreserved in DMSO. 5000 CD4+ T cells were activated with 2500 anti-CD3 and anti-CD28 beads (Dynal beads obtained from Invitrogen, Life Technologies, Carlsbad, USA) for 5 days in the presence of 5000 CD56^bright/dim^ cells previously cultured with or without IL-27. CD4+ T cells alone and CD56^bright/dim^ cells alone were used as controls. Cells were pulsed with ^3^H-thymidine (1 µCi/well) for 16–24 hrs at the end of the incubation period.

### Statistical analysis

The following statistic tests were used: two-tailed paired t-test, two tailed Wilcoxon matched-pairs test, Kruskal-Wallis test with Dunn's multiple comparisons test. A p≤0.05 was considered significant.
